# Exogenous myo-inositol enhances drought tolerance in maize seedlings by antioxidant defense, and photosynthetic efficiency

**DOI:** 10.3389/fpls.2025.1609338

**Published:** 2025-06-25

**Authors:** Yuqi Liu, Hao Sun, Xiaonan Guo, Tianyuan Song, Zongling Yu, Wei Li, Yaxin Lin, Yanci Zhou, Deguang Yang

**Affiliations:** ^1^ Key Laboratory of Germplasm Enhancement, Physiology and Ecology of Food Crops in Cold Region, Ministry of Education, Northeast Agricultural University, Harbin, China; ^2^ College of Resources and Environment, Northeast Agricultural University, Harbin, Heilongjiang, China; ^3^ National Key Laboratory of Smart Farm Technologies and Systems, Northeast Agricultural University, Harbin, China; ^4^ Heilongjiang Provincial Ecological Environment Monitoring Center, Harbin, Heilongjiang, China

**Keywords:** drought stress, maize, seedlings, exogenous myo-inositol, osmotic adjustment

## Abstract

**Introduction:**

Drought stress severely impairs maize (*Zea mays* L.) production, particularly during the seedling stage.

**Methods:**

In this study, we aimed to investigate the role of exogenous myo-inositol (MI) in alleviating drought stress in maize seedlings. We established four treatments: control (CK), MI application under normal irrigation (G), drought stress (D), and MI application under drought stress (DG).

**Results:**

The results demonstrate that MI significantly restored growth parameters under drought conditions, increasing shoot and root biomass by 40.74% and 28.30%, respectively, on Day 7. Additionally, MI enhanced photosynthetic efficiency (net photosynthetic rate (Pn), transpiration rate (Tr), stomatal conductance (gs), and photosystem II efficiency (Fv/Fm)) and upregulated osmotic regulators (e.g. proline and soluble sugars) in the leaves and roots. Furthermore, antioxidant enzymes (superoxide dismutase (SOD), peroxidase (POD), and glutathione reductase (GR)) and ascorbic acid–glutathione (ASA–GSH) cycle components were synergistically activated by MI, reducing oxidative damage, as indicated by decreases in malondialdehyde (MDA), H_2_O_2_, and O_2_^·-^. Principal component analysis highlighted the pivotal roles of osmotic adjustment and antioxidant systems in drought mitigation.

**Discussion:**

These findings reveal that MI is a potent inducer of drought resilience in maize, offering a novel strategy for maize cultivation under water scarcity.

## 1 Introduction

Drought is a key abiotic stressor that constrains global maize (*Zea mays* L.) production and has considerable detrimental effects on seedlings. Maize seedlings at the three-leaf stage exhibit heightened sensitivity to water deficits, which is primarily attributed to their underdeveloped root systems and immature stomatal regulation mechanisms (Yu et al., 2024). Drought-induced osmotic stress impairs maize growth and development, reduces photosynthetic efficiency, elevates oxidative stress levels, and disrupts osmotic homeostasis, collectively contributing to substantial yield losses (He et al., 2024). Although plants establish a foundational defence network through osmoprotectant synthesis (e.g. proline and soluble sugars) and antioxidant enzyme activation (e.g. superoxide dismutase (SOD) and catalase (CAT)), endogenous synthesis rates in seedlings often fail to counteract stress-induced damage because of their immature metabolic capacity (Moharramnejad et al., 2016). Thus, exogenous induction strategies have emerged as promising approaches for enhancing drought resilience in maize seedlings (Zhang et al., 2023; Wang et al., 2024). In particular, the exploration of exogenous inducers for the rapid relief of drought in maize seedlings is important for improving drought tolerance at the seedling stage and securing maize yields.

Exogenous inducers such as salicylic acid, abscisic acid, and polyamines are externally applied chemicals and biological reagents that regulate plant physiological and metabolic processes and increase plant tolerance to stress (Saruhan et al., 2012; Roghayyeh et al., 2014). Among the diverse exogenous inducers, myo-inositol (MI) has emerged as a compound of particular interest because of its dual mechanistic roles, functioning both as an important component of cell membrane phospholipids, participating in phosphatidylinositol synthesis and acting as an osmoprotectant to stabilise the integrity of cell membranes by decreasing the production of markers of oxidative damage, such as MDA, and as a pivotal signalling intermediate in the phosphatidylinositol pathway to modulate stomatal aperture dynamics (Gillaspy, 2011). It has been shown that the application of exogenous MI substantially reduces H_2_O_2_ and mill damage in pepper (*Piper nigrum* L.) leaves under drought stress (Yildizli et al., 2018). Under salt stress conditions in quinoa (*Chenopodium quinoa* Willd.), MI treatment attenuates oxidative injury through upregulation of antioxidant enzymes (e.g. SOD and CAT) and stabilization of membrane integrity (Al-Mushhin et al., 2021). Similarly, maize subjected to salinity stress exhibits improved photosynthetic efficiency, restored reactive oxygen species (ROS) homeostasis, enhanced osmotic adjustment, and optimised nutrient uptake following MI application, collectively counteracting the detrimental effects of salt toxicity (Fatima et al., 2024). 20 Especially at the seedling stage, which is a stress-sensitive period, early spring droughts are especially likely to cause irreversible damage to graminaceous crops and thus affect crop yields; however, it is unclear whether MI can effectively alleviate drought stress in maize seedlings.

Therefore, in this study, we aimed to explore the response of maize seedlings to exogenous MI application. To this end, we used 15% polyethylene glycol (PEG-6000) to simulate drought stress in maize seedlings and applied four different treatments: normal irrigation (CK), normal irrigation with exogenous MI (G), drought stress (D), and drought stress with exogenous MI (DG). The main objective of this study was to determine: (1) whether MI mediates a synergistic mechanism involving osmoregulation (e.g. soluble sugars and proline) and antioxidant defence (e.g. SOD, ascorbate peroxidase (APX), and glutathione reductase (GR)) and (2) whether exogenous MI affects photosynthetic performance and biomass allocation patterns, thereby influencing the growth performance of maize seedlings. The results of this study provide key theoretical support for the innovation of drought-resistant maize cultivation technology and a scientific basis for the effect of exogenous MI on crop drought resistance at seedling stage.

## 2 Materials and methods

### 2.1 Experimental design

The experiment was conducted at the Crop Physiology Laboratory of Northeast Agricultural University (Harbin, China; 126°73′E, 45°74′N). Seeds of maize cultivar ‘Zhengdan 958 (widely adapted varieties mainly grown in northern China, effective accumulated temperature = 2945°C·d)’ were sterilised with 1% NaClO for ten minutes, germinated at 28°C, and transplanted into pots (50 × 38 × 20 cm) filled with vermiculite. Seedlings were grown in a controlled environment (16 h light/8 h dark, 400 μmol·m^−^²·s^−^¹ light intensity, 25°C, 70% humidity). Four treatments were applied: (1) CK, Hoagland’s solution + foliar spray of distilled water; (2) G, Hoagland’s solution + foliar spray of 800 mg·L^−^¹ MI; (3) D, Hoagland’s solution with 15% PEG-6000 (*ψ* = -0.20 MPa) (Michel and Kaufmann, 1973) + distilled water spray; and (4) DG, Hoagland’s solution with 15% PEG-6000 (*ψ* = -0.20 MPa) + 800 mg·L^−^¹ MI spray. Each treatment was replicated five times for a total of 160 seedlings in 20 pots to simulate osmotic stress treatments at the three-leaf stage (Shereen et al., 2022). The PEG-6000 solution was prepared using Hoagland nutrient solution. Hoagland complete nutrient solution (3.78 g·kg^-1^) was applied to the corn seedlings every other day at 1 L per pot. In addition, the leaves of the corn seedlings were sprayed with an 800 mg·L^-1^ solution of either MI or distilled water at a volume of 80 mL per pot, according to the previously published application dosage (Song et al., 2019), for 7 d. After the onset of osmotic stress treatment, samples were collected at 1 d, 3 d, 5 d, and 7 d to measure physiological parameters. Samples were stored in a refrigerator at -80°C for further analysis.

### 2.2 Growth parameters

After 7 d of treatment, the maize seedlings were subjected to enzyme deactivation at 105°C for 30 min, then dried at 80°C to a constant weight and weighed. We calculated the relative effects of the treatments by comparing the change in dry matter between the treatments and control. The dry mass accumulation rate (*DMR*) was calculated as follows:


$$DMR(\%)=\frac{A_{1}-A_{0}}{A_{0}}\times 100$$

where A_1_-A_0_ represents the respective dry matter masses of the latter day minus that of the previous day (For example, when A_1_ is the weight of dry matter on the third day, A_0_ is for the first day; when A_1_ is the weight of dry matter on the fifth day, A_0_ is for the third day, and so on.).

The root:shoot ratio was calculated as follows:


$$Root:shoot\;ratio=\frac{Dry\;weight\;of\;root}{Dry\;weight\;of\;shoot}\;$$

### 2.3 Photosynthesis and chlorophyll fluorescence

We measured the net photosynthetic rate (Pn), photosystem II efficiency (Fv/Fm), transpiration rate (Tr), and stomatal conductance (gs) of the second expanded leaf of each plant using a CI-340 handheld photosynthesis measurement system (CID, USA). The light intensity in the leaf chamber was 400 μmol·m^−^²·s^−^¹ at 25°C and 65% humidity. After 20 min of dark treatment, chlorophyll fluorescence parameters were measured using a plant light efficiency analyser (Pocket Pea, Hansatech, UK). We measure between 9 and 10 a.m. every day.

### 2.4 Content of osmoregulatory substances

We determined the proline content in the maize leaves and roots using the sulfosalicylic acid method. Determination of soluble sugars and sucrose in the maize leaves and roots was performed using anthrone colourimetry (Lu et al., 2024; Yang et al., 2024). The fructose levels in the maize leaves and roots were determined using the resorcinol colourimetric method (Garrett and Young, 1969).

### 2.5 Oxidizing substance content and antioxidant enzyme activity

We measured the malondialdehyde (MDA), O_2_^·−^, H_2_O_2_, SOD, peroxidase (POD), and CAT activities in the maize leaves and roots using commercial kits (Beijing Sunshine Biotechnology Co., Ltd., China), according to the manufacturer’s instructions. The ascorbic acid (ASA), APX, dehydroascorbate reductase (DHAR), monodehydroascorbate reductase (MDHAR), and GR activities and glutathione (GSH) content in the maize leaves and roots were measured using appropriate kits (Jiangsu Addison Biotechnology Co., Ltd., China). Biochemical measurements were expressed per gram of fresh weight (FW).

### 2.6 Data analysis

The experimental data were analysed using SPSS 27.0 software for data collation, principal component analysis of the experimental data, and the test of normal distribution and equality of variance for all data. Data were compared using two-way ANOVA, and when the interaction between factors was significant, one-factor analysis of variance (ANOVA) and least significant difference (LSD) test was performed. Significant differences between means were tested using two-way ANOVA (p < 0.05), and graphs were plotted using Origin 2021.

## 3 Results

### 3.1 Maize seedling growth

**Figure 1:**
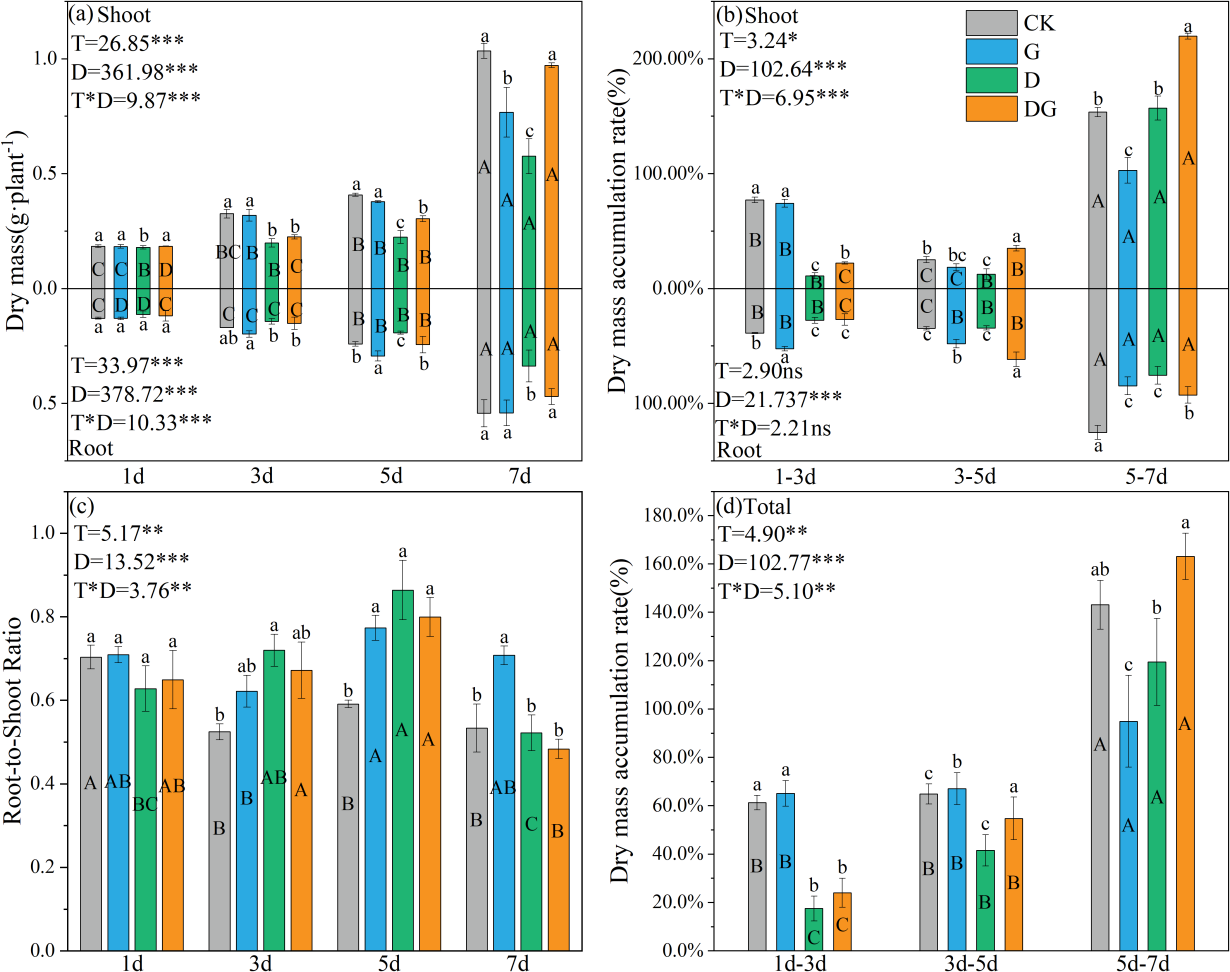
Growth parameters of maize seedlings under different treatments. **(a)** Effect of dry mass of maize seedlings. **(b)** Effect of dry mass accumulation of maize seedlings. **(c)** Effect of root:shoot ratio of maize seedlings. **(d)** Effect of total dry mass accumulation of maize seedlings. CK: no stress; G: only spraying myo-inositol; D: only osmotic stress; DG: osmotic stress and spraying myo-inositol. Lowercase letters (a–d) represent significance between treatments at the same time period, while uppercase letters (A–D) represent significance before the same treatment at different time periods. T stands for treatment, D stands for days, and T*D stands for the interaction of treatment and days. There was no significant difference in the mean of the same letters when P ≥ 0.05.

Two-way ANOVA revealed that treatment, duration of treatment, and their interaction exerted significant effects on both the accumulation amounts and rates of shoot and root dry matter in maize plants, with the exception of the shoot dry matter accumulation rate (**Figure 1**). From 3 d onward, treatment group D demonstrated significantly reduced shoot and root biomass accumulation compared with that of the control group, with decreases of 44.35% and 37.94%, respectively, observed by 7 d (**Figure 1a**). In contrast to those in treatment group D, plants in treatment group DG exhibited significantly enhanced biomass accumulation starting from 5 d, showing 40.74% and 28.30% increases in shoot and root biomass, respectively, by 7 d (**Figure 1b**). Furthermore, both treatment groups D and DG displayed significantly elevated root:shoot ratios compared with that of the control group during Days 3–7. Specifically, treatment group DG showed 22.30% and 26.41% increases in the root:shoot ratio at 3 d and 5 d, respectively, compared with that of the control group, and this difference was statistically insignificant by 7 d (**Figure 1c**). Regarding biomass accumulation rates, distinct patterns emerged: treatment groups CK and G displayed an initial decline followed by a rapid increase in shoot accumulation rates, whereas treatment groups D and DG maintained progressive increases in both shoot and root accumulation rates. Notably on Days 5–7, treatment group DG exhibited significantly higher shoot dry matter accumulation rates than the control group.

### 3.2 Leaf photosynthesis

**Figure 2:**
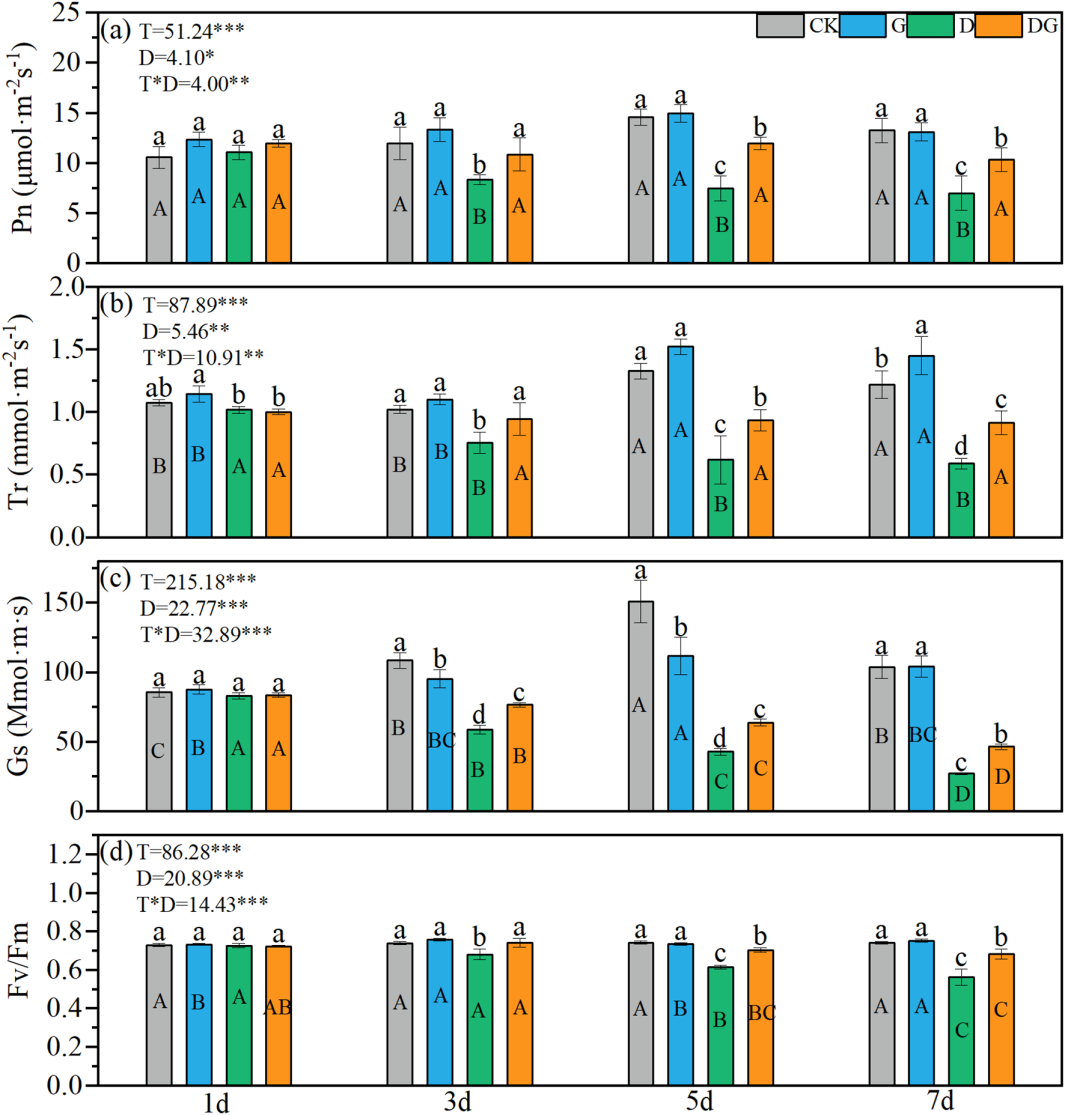
Photosynthetic capacity of maize seedlings under different treatments. Effect of net photosynthetic rate **(a)**, transpiration rate **(b)**, stomatal conductance **(c)**, Fv/Fm **(d)** of maize seedlings. Lowercase letters (a-d) represent significance between treatments at the same time period, while uppercase letters (A, B, C) and (D) represent significance before the same treatment at different time periods. T stands for treatment, D stands for days, and T*D stands for the interaction of treatment and days. There was no significant difference in the mean of the same letters when P ≥ 0.05.

Two-way ANOVA revealed significant effects of treatment, duration of treatment, and their interaction on maize photosynthetic parameters (Pn, Tr, Gs, and Fv/Fm) (**Figures 2a–d**). Treatment group D exhibited decreasing trends in all four parameters over time, with values significantly lower than those of the control group. By 7 d, reductions of 30.21%, 26.05%, 45.61%, and 52.38% were observed for Pn, Tr, Gs, and Fv/Fm, respectively, compared with those of the control group (**Figures 2a–d**). In contrast, treatment group DG showed significant increases in Pn, Tr, Gs, and Fv/Fm of 47.57%, 55.37%, 70.61%, and 21.54%, respectively, compared with those of treatment group D at 7 d, although the values remained significantly lower than those of the control group (**Figures 2a–d**). Notably, treatment group DG maintained stable Pn and Tr values throughout the experimental period, whereas its Gs and Fv/Fm values exhibited decreasing trends over time.

### 3.3 Proline and sugar content of maize seedlings

**Figure 3:**
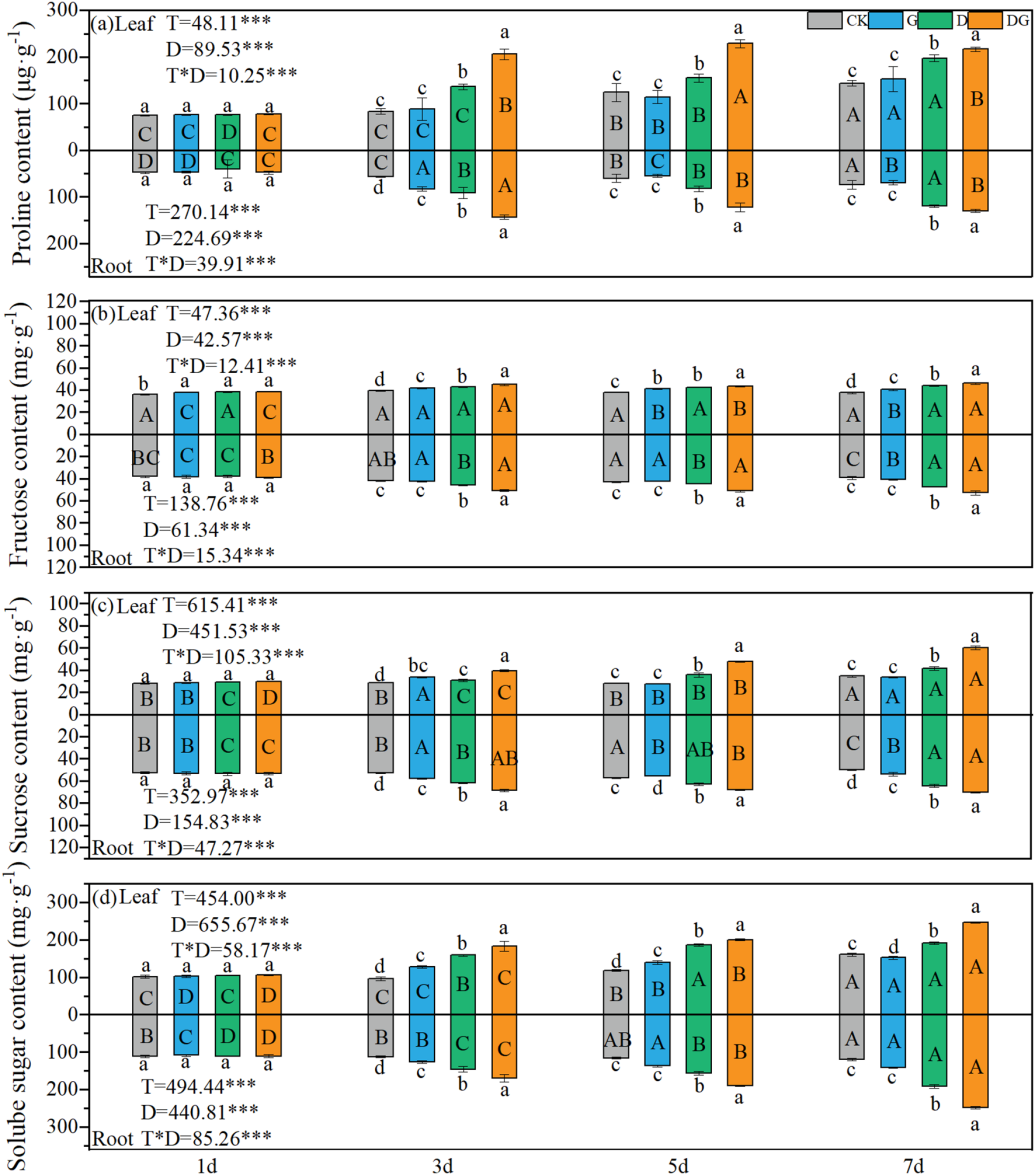
Proline and sugar content in maize seedlings under different treatments. Effect of proline content **(a)**, Fructose content **(b)**, Sucrose content **(c)** and Solube sugar content **(d)** in leaf and root of maize seedlings. Lowercase letters (a–d) represent significance between treatments at the same time period, while uppercase letters (A–D) represent significance before the same treatment at different time periods. T stands for treatment, D stands for days, and T*D stands for the interaction of treatment and days. There was no significant difference in the mean of the same letters when P ≥ 0.05.

Two-way ANOVA revealed highly significant effects (p < 0.001) of treatment, duration of treatment, and their interaction on the proline, sucrose, fructose, and soluble sugar contents in both the leaves and roots of maize plants. Treatment group D demonstrated increasing trends in all measured parameters (proline, sucrose, fructose, and soluble sugars) across both tissues over time, with values consistently surpassing those of the control group (**Figures 3a–d**). In treatment group DG, while sucrose and soluble sugars maintained upward trends in the leaves and roots, the leaf proline content showed a significant decline at 7 d compared with that at 5 d. Whereas root proline exhibited a transient elevation on Day 3, followed by a reduction, and leaf fructose displayed a temporary decrease on Day 5 before making a subsequent recovery (**Figures 3a, c, d**). Compared with CK, treatment group D induced 37.26% and 18.77% increases in leaf proline and soluble sugar contents, respectively, on Day 7 (**Figures 3a, d**). Furthermore, treatment group DG enhanced leaf proline, fructose, sucrose, and soluble sugar contents beyond treatment group D levels, with proline showing 51.40%, 47.46%, and 9.69% increases at 3 d, 5 d, and 7 d, respectively, and soluble sugars attaining a 29.32% elevation at 7 d compared with the level under treatment group D (**Figures 3a, d**). In root tissues, treatment group D elevated the proline and soluble sugar contents by 62.52% and 59.79%, respectively, at 7 d compared with those under CK (**Figures 3a, d**). Treatment group DG further increased the proline and soluble sugar contents in the root system by 8.42% and 29.76%, respectively, at 7 d compared with those under treatment D (**Figures 3a, d**).

### 3.4 Lipid peroxidation product of maize seedlings

**Figure 4:**
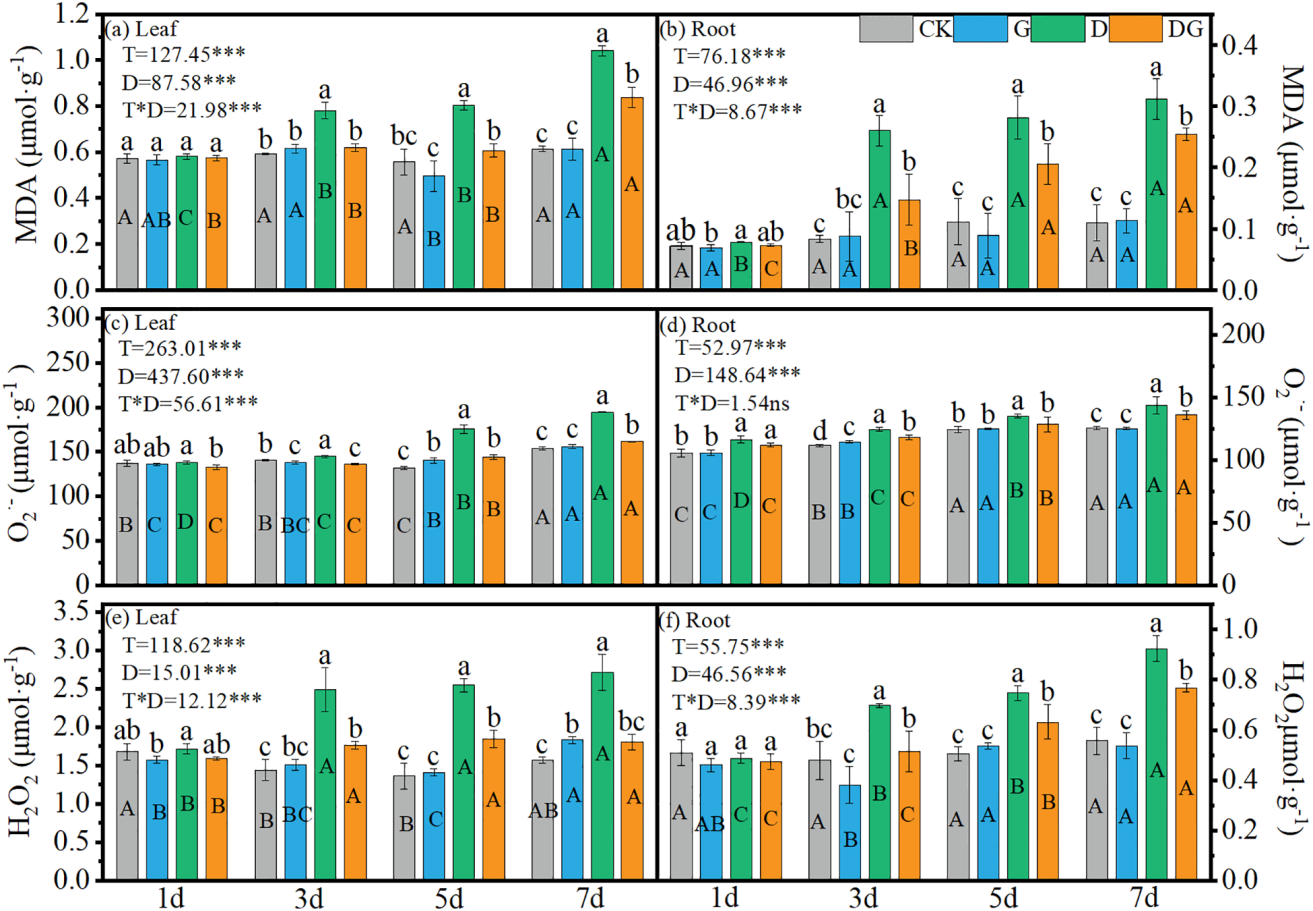
Lipid peroxidation product in maize seedlings under different treatments. Effect of MDA content **(a, b)**, O_2_^·^−^^ content **(c, d)**, H_2_O_2_ content **(e, f)** in leaf and root of maize seedlings. Lowercase letters (a–d) represent significance between treatments at the same time period, while uppercase letters (A–C) and (D) represent significance before the same treatment at different time periods. T stands for treatment, D stands for days, and T*D stands for the interaction of treatment and days. There was no significant difference in the mean of the same letters when P ≥ 0.05.

Two-way ANOVA revealed highly significant (p < 0.001) effects of treatment, duration of treatment, and their interaction on the MDA, O_2_^·−^ and H_2_O_2_ contents in the maize leaves and roots, except for the non-significant interaction effect on root O_2_^·−^ concentration (**Figures 4a–d**). Treatment groups D and DG exhibited increasing trends in MDA, O_2_^·−^, and H_2_O_2_ contents across tissues over time. Compared with CK, treatment group D induced 69.52% and 72.64% increases in leaf MDA and H_2_O_2_ contents, respectively, at 7 d (**Figures 4a, e**). Similarly, in roots, treatment group D elevated the MDA and H_2_O_2_ contents by 183.04% and 65.99%, respectively, at 7 d compared with those under CK (**Figures 4b, f**). Notably, compared with treatment group D, treatment DG significantly attenuated these oxidative markers, achieving 19.35% and 33.54% reductions in leaf MDA and H_2_O_2_, respectively, along with 18.45% and 17.01% decreases in root MDA and H_2_O_2_, respectively, at 7 d (**Figures 4a–f**).

### 3.5 Antioxidant activities of maize seedlings

**Figure 5:**
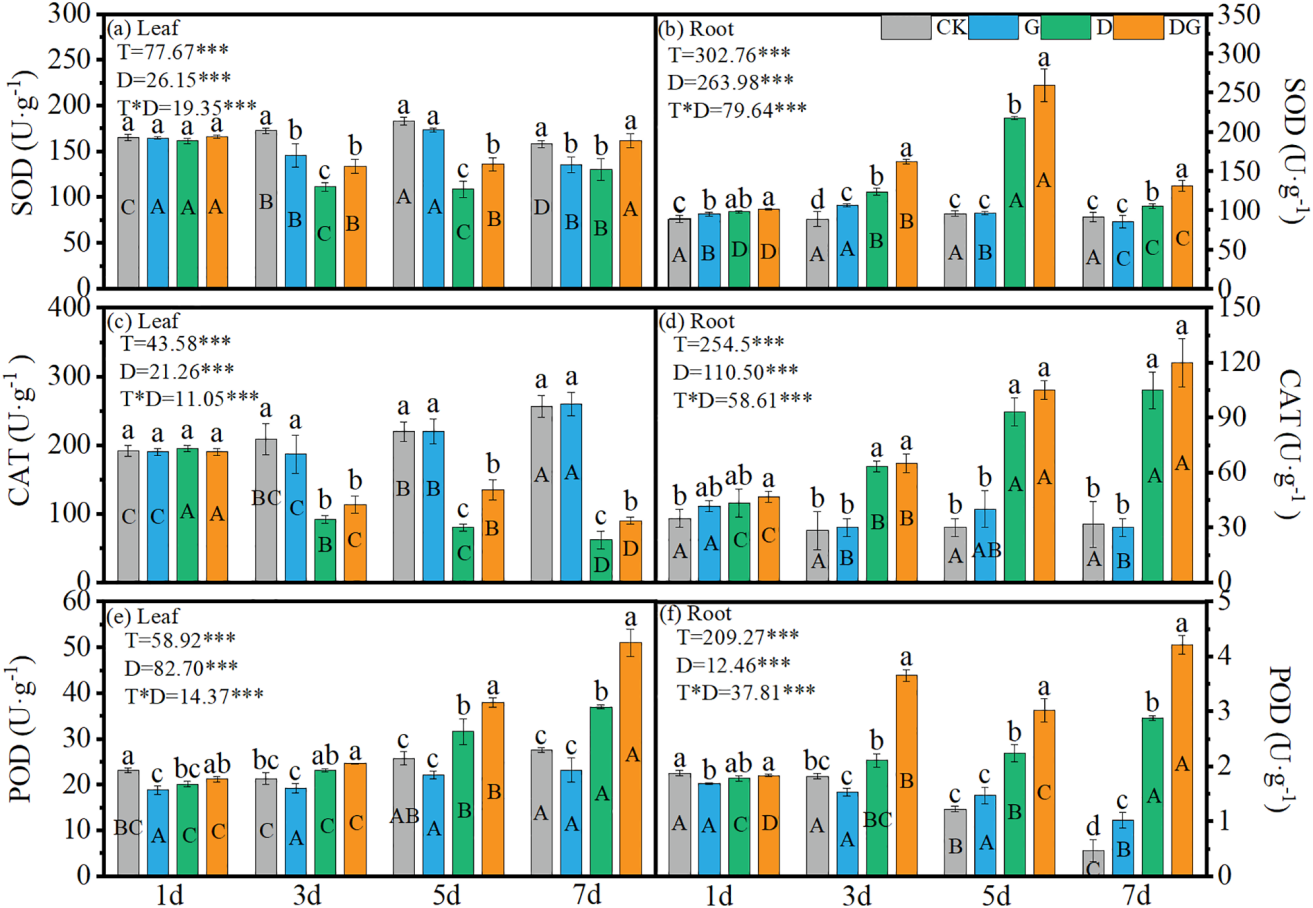
Antioxidant enzyme activities in maize seedlings under different treatments. Effect of SOD activity **(a, b)**, CAT activity **(c, d)**, POD activity **(e, f)** in leaf and root of maize seedlings. Lowercase letters (a-d) represent significance between treatments at the same time period, while uppercase letters (A-D) represent significance before the same treatment at different time periods. T stands for treatment, D stands for days, and T*D stands for the interaction of treatment and days. There was no significant difference in the mean of the same letters when P ≥ 0.05.

Two-way ANOVA revealed highly significant (p < 0.001) effects of treatment, duration of treatment, and their interaction on the SOD, CAT and POD activities in the maize leaves and roots. In the leaves, treatment groups D and DG showed decreasing SOD trends from 1 d to 5 d, followed by significant activation after 5 d (**Figure 5a**). In contrast, in the roots, the SOD activity initially increased during Days 1–5 and then declined, with treatment D achieving 127.26% higher activity than CK at 5 d, while treatment group DG attained a further 19.21% increase over treatment group D (**Figure 5b**). Leaf CAT activity progressively declined in the D and DG treatment groups, remaining below that in the control group, whereas root CAT showed inverse patterns, with 231.58% and 278.95% increases in treatment groups D and DG, respectively, compared with that under CK at 7 d (**Figures 5c, d**). POD activities showed upward trends in the D and DG treatment groups across tissues, although the control group exhibited decreasing root POD activity (**Figures 5e, f**). At 7 d, leaf POD activity increased by 34.11% under treatment group D compared with that under CK, with treatment group DG achieving a 37.76% enhancement over the level under treatment group D. Root POD demonstrated more pronounced effects: a 512.77% increase under treatment group D and a 46.18% additional elevation under treatment group DG compared with that under treatment group D (**Figures 5e, f**).

### 3.6 ASA-GSH cycle in maize seedlings

**Figure 6:**
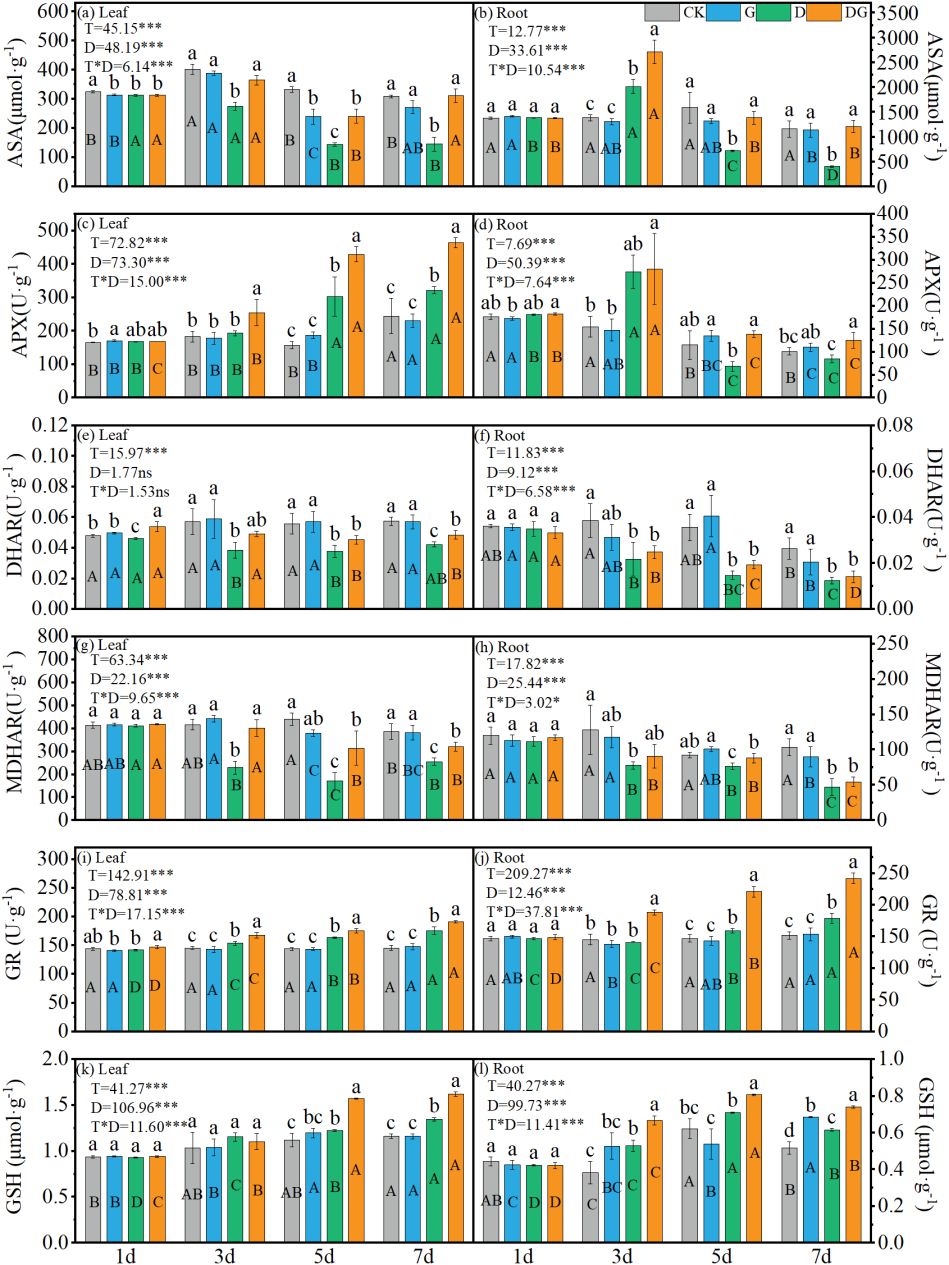
ASA-GSH cycle in maize seedlings under different treatments. Effect of ASA content **(a, b)**, APX activity **(c, d)**, DHAR activity **(e, f)**, MDHAR activity **(g, h)**, GR activity **(i, j)**, GSH content **(k, l)** in leaf and root of maize seedlings. Lowercase letters (a-d) represent significance between treatments at the same time period, while uppercase letters (A-D) represent significance before the same treatment at different time periods. T stands for treatment, D stands for days, and T*D stands for the interaction of treatment and days. There was no significant difference in the mean of the same letters when P ≥ 0.05.

Two-way ANOVA revealed significant (p < 0.05) effects of treatment, duration of treatment, and their interaction on the ASA and GSH content and APX, DHAR, MDHAR and GR activities in maize leaves and roots. However, days and their interaction showed no significant influence (p > 0.05) on leaf DHAR activity. Under treatment group D, the ASA content showed decreasing trends in both tissues, although a transient increase was observed in the roots at 3 d. By 7 d, treatment group D caused significant reductions of 56.57% and 52.97% in the ASA content in the leaves and roots, respectively, compared with that under CK (**Figures 6a, b**). Treatment DG effectively reversed this pattern, increasing the ASA content in the leaves and roots by 67.08% and 113.76%, respectively, at 7 d compared with that under treatment group D, reaching levels comparable to those under CK (**Figures 6a, b**). Meanwhile, APX activity under treatment groups D and DG exhibited tissue-specific responses: progressive increases in the leaves contrasted with initial decreases followed by transient activation in the roots. Both the DHAR and MDHAR activities displayed consistent declines under the treatment groups D and DG across tissues, remaining below the CK levels throughout the experimental period. Although treatment group DG moderately improved these enzymatic activities compared with those under treatment group D, they were not restored to CK levels (**Figures 6e–h**). In contrast, both GR activity and GSH content in leaves and roots under treatment groups D and DG showed an increasing trend with time, and all of them were significantly higher than those of CK after 5 d (**Figures 6i–l**). The GR activity in the leaves under treatment groups D and DG was significantly increased by 20.30% and 31.47%, respectively, at 7 d compared with that under CK, whereas the GR content in the roots was significantly increased by 17.62% and 59.28% under treatments D and DG, respectively, at 7 d (**Figures 6i, j**).

### 3.7 Principal component analysis

Principal component analysis at 3 d revealed a distinct separation between leaf and root systems, with the first two principal components explaining 76.6% (PC1: 49.6%; PC2: 27.0%) of the total variance (**Figure 6a**). Temporal analysis revealed dynamic parameter associations: dry matter accumulation, antioxidant enzymes, and osmolytes exhibited strong positive correlations with PC1 during Days 5–7, while photosynthetic characteristics displayed contrasting patterns, with initially negative loadings on PC2 on Day 3 that transitioned to positive loadings by Days 5–7 (**Figures 6a–c**). These loading patterns underscore the critical regulatory roles of these physiological parameters in maize seedling development. The variance distribution evolved significantly over time. At 5 d, PC1 and PC2 accounted for 75.8% of the cumulative variance (42.8% and 33.0%, respectively), with overlapping organ clusters. This contrasted with the 7 d results, where the components explained 80.4% of the variance (PC1: 47.5%; PC2: 32.9%), accompanied by renewed tissue-specific differentiation. The progressive expansion of PC2’s explanatory power from 27.0% to 32.9% between sampling points suggests the increasing importance of photosynthetic regulation during the later growth stages.

## 4 Discussion

### 4.1 Regulation of photosynthetic performance and biomass allocation by myo-inositol during maize seedling growth

Drought stress impedes maize seedling growth by suppressing photosynthetic activity and metabolic processes, thereby reducing biomass accumulation (Zhang et al., 2015). In our study, exposure to drought stress significantly reduced key photosynthetic parameters, including Pn, Tr, Gs, and Fv/Fm. However, several studies have indicated that exogenous MI application effectively mitigates these adverse effects (Li et al., 2020). In our study, although the dry matter accumulation following MI treatment under drought stress was still reduced compared with that under the control before 7 d, there was no significant difference from the control at 7 d, and the shoot and root dry matter accumulation increased by 40.74% and 28.30%, respectively, compared with that under drought stress without MI treatment. This was consistent with the trend in Pn values, which were reduced under drought stress but recovered following MI application (**Figures 1a**, **2a**). Especially during Days 5– 7 of the treatment period, the application of MI effectively increased the rate of dry matter accumulation in seedlings under drought stress (**Figure 1**), indicating its capacity to restore growth under osmotic stress (Fatima et al., 2024). Early stress exposure resulted in a significantly increased root:shoot ratio in MI-treated plants (**Figure 1**), suggesting enhanced resource allocation to the root systems (Lange, 2023). Although MI supplementation significantly improved the Pn, Gs, and Tr compared with those of untreated drought-stressed plants, these parameters remained below the CK levels (**Figure 2**). This suggests that MI may act as a signalling molecule in stress adaptation, participating in the regulation of stomatal movement to maintain the CO_2_ concentration and enhance photosynthetic efficiency (Gillaspy, 2011). The transient increase in the root:shoot ratio after inositol application reflects the dynamic balance between root growth and shoot photosynthetic efficiency under drought stress conditions, which is a key strategy for drought tolerance in maize (Sharma et al., 2019). Furthermore, MI treatment significantly increased the soluble sugar content and elevated both the dry matter accumulation rate and total accumulation compared with that of untreated drought-stressed plants, although the accumulation remained below the CK levels (**Figures 1**, **3**). This suggests that MI facilitates carbon source provision for biomass accumulation through enhanced sugar synthesis and transport, potentially by redirecting photosynthetic products towards the synthesis of carbon assimilates rather than plant growth (Sharma et al., 2019; Jarin et al., 2024). These findings support the likelihood of coordinated sugar metabolism and growth regulation (Wu et al., 2025). Overall, our results demonstrate that exogenous MI application partially restores drought-impaired photosynthetic capacity and modifies resource allocation patterns in maize seedlings.

### 4.2 Myo-inositol raises proline and sugar content in maize seedlings

Osmotic adjustment is a critical mechanism for maintaining cellular turgor pressure in plants under drought conditions. In maize seedlings under drought stress, the osmotic balance is typically maintained through the accumulation of osmolytes such as proline and total soluble sugars (**Figure 3**) (Ghosh et al., 2021; Rasheed et al., 2024). Our results reveal that while photosynthetic capacity decreased under drought stress, total soluble sugar accumulation significantly increased compared with that under control conditions. This phenomenon may be attributed to the MI-mediated redirection of dry matter allocation towards osmotic adjustment processes (Sharma et al., 2019; Jarin et al., 2024), thereby substantially enhancing the accumulation of osmoregulatory substances, including proline and carbon assimilates, in both the leaves and roots (**Figures 1**–**3**). These results are consistent with those for Arabidopsis and rice, suggesting that MI can upregulate the synthesis of osmoregulatory substances under abiotic stress and strengthen the osmotic adaptation of roots by modulating sugar metabolism pathways (Pamuru et al., 2021). Notably, the role of inositol in raising proline and sugar is not limited to enhancing osmoregulation. It has also been demonstrated that MI improves osmoregulatory capacity by stabilising membrane structures, raising proline content and reducing ion leakage (Gurrieri et al., 2020; Renzetti et al., 2024). Our results corroborate this mechanism; exogenous MI application under drought stress induced a significant early stage increase in the root:shoot ratio and substantially reduced the ROS content compared with that of untreated drought-stressed plants (**Figures 1**, **4**). It shows that MI mitigates drought damage to cells by increasing dry matter partitioning to the root system, activating the ROS elimination program, and mitigates drought damage to cells in conjunction with osmoregulatory substances. Through membrane integrity stabilisation and metabolic pathway modulation, MI possibly maintains osmotic equilibrium, delays cellular dehydration, and preserves metabolic activity during prolonged drought exposure.

### 4.3 Myo-inositol regulates redox homeostasis by activating the antioxidant system of maize seedling leaves

Drought-induced ROS accumulation is the primary cause of oxidative damage (Samanta et al., 2024). In this study, oxidative markers (MDA, H_2_O_2_, and O_2_^·−^) in both leaves and roots were significantly elevated under drought stress by 3 d compared with control levels. Exogenous MI application markedly reduced these oxidative indicators relative to those in untreated drought-stressed plants, although the levels remained elevated compared with those in non-stressed controls (**Figure 4**), which may be attributed to incomplete recovery within the 7 d treatment period. Drought-triggered oxidative stress was substantially alleviated by applying exogenous MI, and this mitigation correlated closely with the enhanced activity of key antioxidant enzymes, including SOD, POD, GR, and APX (**Figure 5**). The coordinated upregulation of these enzymes demonstrates that MI reinforces the ROS-scavenging network to protect the photosynthetic apparatus from oxidative injury, which is consistent with previous findings (Jia et al., 2019). In this study, CAT activity in the leaves was inhibited under drought stress and remained significantly lower than that in the control, although it was partially restored upon application of exogenous MI. However, CAT activity in the roots was significantly higher under drought stress than that in the control, suggesting that the regulation of the antioxidant system may be more dependent on SOD and POD in leaves and on CAT and POD in roots (**Figure 5**). This may be because the root system is the first to be affected in drought stress and is the key organ for water uptake, whereas the leaves have a relatively low demand for CAT through stomatal regulation and metabolic inhibition to reduce ROS production. These results emphasise the important role of MI in regulating interorgan redox homeostasis, which is a key factor in maintaining plant growth under stress (Islam et al., 2020).

Meanwhile, the synergistic regulation of the ASA–GSH cycle represents another central mechanism through which MI alleviates oxidative stress, and the application of MI significantly restored the ASA content and APX activity in leaves under drought stress (Zhou et al., 2024). Meanwhile, in the root system, changes in ASA content and APX, DHAR and MDHAR activities were consistent with previous findings (**Figure 6**) (Mellidou and Kanellis, 2023). This may be because the ASA-GSH cycle is a high energy-consuming pathway, whereas the root system may reduce the ASA-GSH cycle under drought stress due to the reduction of photosynthetic products, but enough photosynthetically synthesised products can be photosynthesised in the leaves to supply the ASA-GSH cycle (Gill and Tuteja, 2010). Through these findings indicate that MI preferentially strengthens ASA regeneration capacity in leaves, ensuring efficient ROS scavenging through APX-mediated pathways. The limited restoration of DHAR and MDHAR in the roots may reflect tissue-specific metabolic constraints or alternative detoxification mechanisms (Ushimaru et al., 2006). Notably, the synergistic effect between the increase in GSH content and enhanced APX activity further demonstrates the important role of MI in the maintenance of redox homeostasis, which is a key feature of plant drought tolerance (Foyer and Noctor, 2011). Our results demonstrate that the ASA–GSH cycle, one of the most vital antioxidant pathways in plants, significantly amplifies the antioxidant capacity through MI-mediated efficiency improvements, thereby mitigating drought-induced oxidative damage. Our findings reveal that MI maintains the ROS homeostatic balance through the coordinated regulation of ROS production and elimination mechanisms, ultimately reducing oxidative injury.

**Figure 7:**
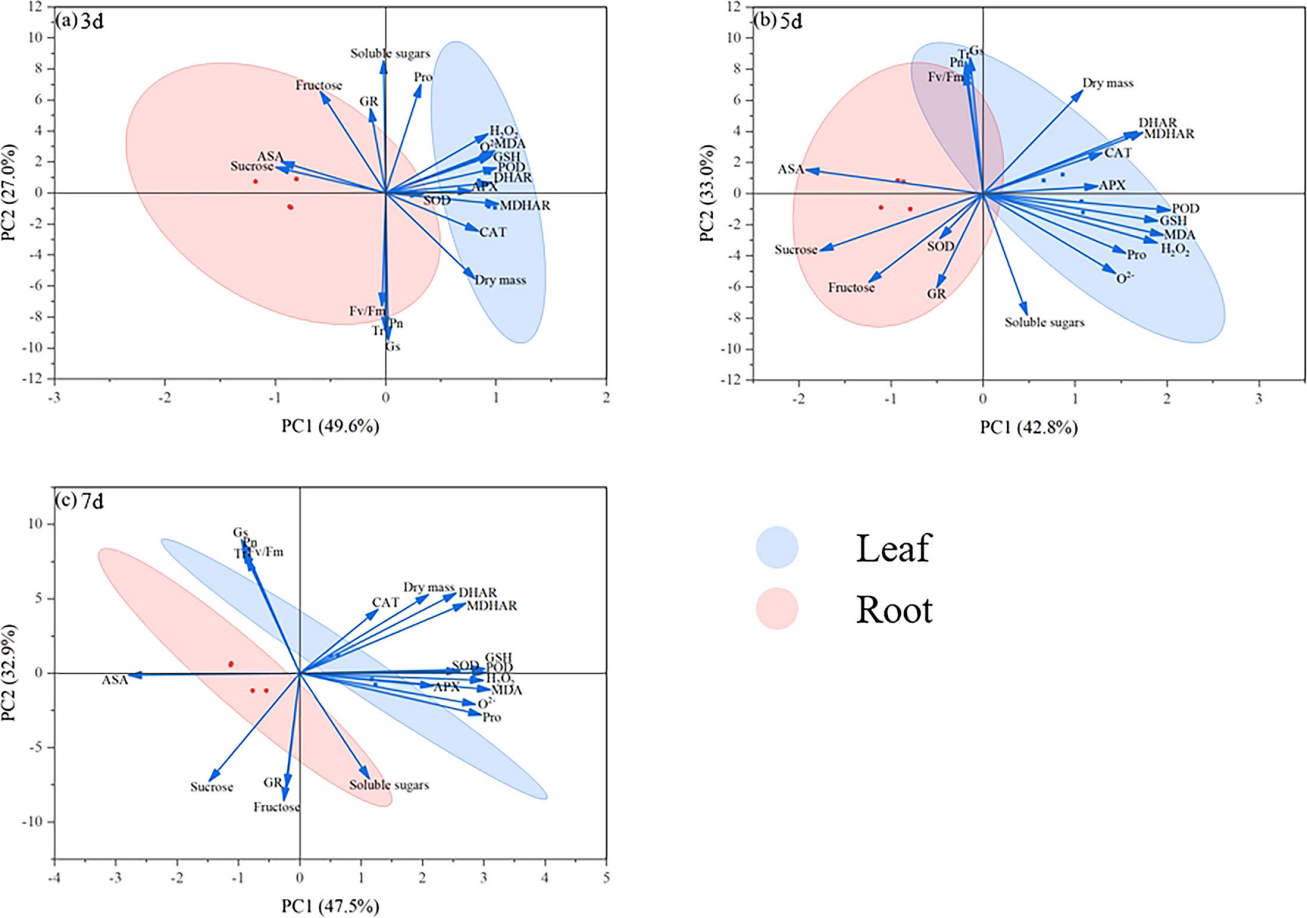
Principal component analysis of leaf and root indicators. The blue represents the treatment of leaves, the pink represents the treatment of roots. State of maize seedling leaves and roots at 3 d **(a)**, 5 d **(b)**, 7 d **(c)**.

Principal component analysis was conducted to systematically explore the multidimensional regulatory effects of exogenous MI in mitigating drought stress in maize seedlings. At 3 d, PC1 and PC2 collectively accounted for 76.6% of the total variance, demonstrating the dominant roles of osmolytes and antioxidant enzyme systems during the initial drought phase, which was consistent with the previous inference, with leaves exhibiting more pronounced responses. Notably, the dry matter and photosynthetic parameters were primarily enriched in the negative PC2 region (**Figure 7a**). As the stress duration progressed to 5 d and 7 d, the contribution rate of PC1 increased to 42.8% and 47.5%, respectively, whereas photosynthetic parameters (Pn and Fv/Fm) showed significant enrichment in the positive PC2 region (**Figures 7b, c**), further indicating that late-stage dry matter accumulation was closely associated with synergistic interactions between osmotic adjustment and antioxidant systems. The PCA results reveal that MI effectively counteracted drought-induced physiological imbalances through the dual enhancement of osmotic regulation capacity and antioxidant defence networks. Our results indicate that exogenous MI alleviates drought-stress effects in maize seedlings via a multidimensional defence strategy involving the coordinated regulation of osmotic homeostasis, antioxidant responses, and photosynthetic efficiency.

## 5 Conclusion

In summary, we concluded that exogenous MI application alleviates the inhibition of photosynthetic performance by maintaining Gs and Fv/Fm; dedicating more assimilates to the maintenance of proline, soluble sugar, and sucrose contents in leaves and roots; and enhancing cellular osmoregulation. It also regulates shoot and root biomass allocation, improves water uptake efficiency by optimising the root:shoot ratio, and significantly alleviates the inhibition of maize seedling growth by drought stress. In addition, MI synergistically increases the activities of antioxidant enzymes such as SOD, POD, and GR and enhances the efficiency of ASA–GSH cycling, which significantly reduces oxidative damage markers, such as MDA, H_2_O_2_, and O_2_^·−^, and maintains redox homeostasis. Myo-inositol is involved in a core pathway that enhances drought tolerance in maize seedlings by integrating osmoregulation, antioxidant defence, and photosynthetic performance restoration to form a multidimensional synergistic mechanism. This study provides a theoretical basis for the application of exogenous MI in improving the drought resistance of maize seedlings. Its application via low-cost and high-efficiency foliar spraying can be used as a novel agricultural technology to help stabilise maize seeding growth in water-scarce areas. However, this paper mainly focuses on the physiological effects of exogenous inositol on maize seedlings, so the relevant metabolic pathways still need to be further explored.

## Data Availability

The original contributions presented in the study are included in the article/**Supplementary Material**, further inquiries can be directed to 15169354122@163.com.
