# Supplementary material for: Exogenous myo-inositol enhances drought tolerance in maize seedlings by antioxidant defense, and photosynthetic efficiency
Source: Front Plant Sci. 2025 Jun 25;16:1609338. doi: 10.3389/fpls.2025.1609338 (PMC12238757; doi:10.3389/fpls.2025.1609338)
Supplement: Supplementary file 1 [file Table1.docx]

|  |  |  | 1 | 2 | 3 |
| --- | --- | --- | --- | --- | --- |
| 3d | Leaf | SOD | -0.909 | 0.077 | -0.409 |
|  |  | POD | 0.798 | 0.288 | -0.529 |
|  |  | CAT | -0.995 | -0.065 | -0.076 |
|  |  | MDA | 0.789 | -0.575 | 0.217 |
|  |  | O₂^·^⁻ | 0.349 | -0.933 | -0.089 |
|  |  | H₂O₂ | 0.895 | -0.431 | 0.119 |
|  |  | GR | 0.725 | 0.605 | -0.329 |
|  |  | GSH | 0.986 | -0.167 | 0.026 |
|  |  | Pro | 0.748 | 0.634 | -0.197 |
|  |  | Fructose | 0.797 | 0.584 | 0.155 |
|  |  | Sucrose | 0.348 | 0.922 | 0.17 |
|  |  | Solublesugars | 0.85 | 0.502 | 0.161 |
|  |  | Drymass | -0.999 | -0.029 | 0.032 |
|  |  | Pn | -0.892 | 0.389 | 0.23 |
|  |  | Tr | -0.895 | 0.411 | 0.175 |
|  |  | Gs | -0.973 | 0.088 | -0.216 |
|  |  | FvFm | -0.756 | 0.646 | 0.101 |
|  |  | ASA | -0.88 | 0.446 | -0.166 |
|  |  | APX | 0.599 | 0.787 | -0.151 |
|  |  | DHAR | 0.442 | 0.319 | 0.838 |
|  |  | MDHAR | -0.897 | -0.021 | 0.442 |
|  | Root | SOD | 0.928 | 0.365 | 0.080 |
|  |  | POD | 0.839 | 0.453 | -0.300 |
|  |  | CAT | 0.987 | -0.162 | 0.010 |
|  |  | MDA | 0.706 | -0.692 | 0.152 |
|  |  | O₂^·^⁻ | 0.755 | -0.570 | 0.325 |
|  |  | H₂O₂ | 0.624 | -0.765 | -0.160 |
|  |  | GR | 0.698 | 0.632 | -0.338 |
|  |  | GSH | 0.825 | 0.438 | 0.358 |
|  |  | Pro | 0.873 | 0.470 | 0.130 |
|  |  | Fructose | 0.948 | 0.311 | -0.068 |
|  |  | Sucrose | 0.947 | 0.276 | 0.164 |
|  |  | Solublesugars | 0.966 | 0.238 | 0.101 |
|  |  | Drymass | -0.800 | 0.418 | 0.430 |
|  |  | Pn | -0.703 | 0.696 | 0.146 |
|  |  | Tr | -0.692 | 0.716 | 0.092 |
|  |  | Gs | -0.843 | 0.438 | -0.312 |
|  |  | FvFm | -0.469 | 0.882 | 0.037 |
|  |  | ASA | 0.961 | 0.218 | -0.170 |
|  |  | APX | -0.916 | -0.297 | -0.271 |
|  |  | DHAR | -0.394 | -0.918 | 0.041 |
|  |  | MDHAR | -0.960 | -0.101 | 0.261 |
| 5d | Leaf | SOD | -0.990 | 0.118 | -0.078 |
|  |  | POD | 0.764 | 0.605 | 0.225 |
|  |  | CAT | -0.977 | 0.098 | -0.188 |
|  |  | MDA | 0.836 | -0.364 | 0.411 |
|  |  | O₂^·^⁻ | 0.856 | -0.513 | 0.067 |
|  |  | H₂O₂ | 0.932 | -0.311 | 0.186 |
|  |  | GR | 0.857 | 0.514 | 0.045 |
|  |  | GSH | 0.550 | 0.800 | -0.238 |
|  |  | Pro | 0.658 | 0.752 | 0.037 |
|  |  | Fructose | 0.841 | 0.245 | -0.482 |
|  |  | Sucrose | 0.719 | 0.695 | -0.003 |
|  |  | Solublesugars | 0.939 | 0.311 | -0.145 |
|  |  | Drymass | -0.977 | 0.204 | -0.054 |
|  |  | Pn | -0.914 | 0.305 | -0.266 |
|  |  | Tr | -0.927 | 0.056 | -0.371 |
|  |  | Gs | -0.985 | 0.025 | 0.169 |
|  |  | FvFm | -0.886 | 0.423 | -0.188 |
|  |  | ASA | -0.861 | 0.431 | 0.271 |
|  |  | APX | 0.956 | 0.290 | -0.035 |
|  |  | DHAR | -0.568 | 0.550 | 0.612 |
|  |  | MDHAR | -0.765 | 0.333 | 0.551 |
|  | Root | SOD | 0.981 | 0.192 | 0.037 |
|  |  | POD | 0.917 | 0.392 | -0.072 |
|  |  | CAT | 0.990 | 0.124 | -0.074 |
|  |  | MDA | 0.925 | -0.366 | 0.099 |
|  |  | O₂^·^⁻ | 0.837 | -0.541 | -0.078 |
|  |  | H₂O₂ | 0.892 | -0.435 | -0.122 |
|  |  | GR | 0.722 | 0.682 | 0.116 |
|  |  | GSH | 0.886 | 0.308 | 0.346 |
|  |  | Pro | 0.850 | 0.511 | 0.131 |
|  |  | Fructose | 0.743 | 0.651 | 0.157 |
|  |  | Sucrose | 0.924 | 0.321 | 0.209 |
|  |  | Solublesugars | 0.868 | 0.444 | -0.221 |
|  |  | Drymass | -0.667 | 0.560 | -0.492 |
|  |  | Pn | -0.840 | 0.542 | -0.026 |
|  |  | Tr | -0.919 | 0.344 | -0.194 |
|  |  | Gs | -0.934 | 0.161 | 0.318 |
|  |  | FvFm | -0.779 | 0.623 | 0.076 |
|  |  | ASA | -0.620 | 0.710 | 0.335 |
|  |  | APX | -0.903 | 0.110 | -0.416 |
|  |  | DHAR | -0.857 | -0.123 | 0.501 |
|  |  | MDHAR | -0.741 | -0.484 | 0.465 |
| 7d | Leaf | SOD | -0.205 | 0.920 |  |
|  |  | POD | 0.776 | 0.627 |  |
|  |  | CAT | -0.993 | -0.068 |  |
|  |  | MDA | 0.961 | -0.225 |  |
|  |  | O₂^·^⁻ | 0.800 | -0.571 |  |
|  |  | H_2_O_2_ | 0.760 | -0.650 |  |
|  |  | GR | 0.897 | 0.438 |  |
|  |  | GSH | 0.768 | 0.635 |  |
|  |  | Pro | 0.931 | 0.352 |  |
|  |  | Fructose | 0.900 | 0.295 |  |
|  |  | Sucrose | 0.681 | 0.728 |  |
|  |  | Solublesugars | 0.740 | 0.672 |  |
|  |  | Drymass | -0.514 | 0.810 |  |
|  |  | Pn | -0.943 | 0.296 |  |
|  |  | Tr | -0.913 | 0.124 |  |
|  |  | Gs | -0.993 | 0.022 |  |
|  |  | FvFm | -0.886 | 0.390 |  |
|  |  | ASA | -0.603 | 0.795 |  |
|  |  | APX | 0.958 | 0.198 |  |
|  |  | DHAR | -0.852 | 0.343 |  |
|  |  | MDHAR | -0.995 | -0.092 |  |
|  | Root | SOD | 0.851 | 0.434 | 0.296 |
|  |  | POD | 0.950 | 0.313 | 0.008 |
|  |  | CAT | 0.992 | 0.090 | 0.090 |
|  |  | MDA | 0.969 | -0.244 | -0.045 |
|  |  | O₂^·^⁻ | 0.925 | -0.374 | -0.066 |
|  |  | H₂O₂ | 0.928 | -0.372 | -0.027 |
|  |  | GR | 0.798 | 0.569 | 0.198 |
|  |  | GSH | 0.503 | 0.706 | -0.498 |
|  |  | Pro | 0.988 | 0.082 | 0.129 |
|  |  | Fructose | 0.944 | 0.328 | 0.045 |
|  |  | Sucrose | 0.962 | 0.267 | -0.059 |
|  |  | Solublesugars | 0.913 | 0.407 | 0.012 |
|  |  | Drymass | -0.818 | 0.556 | 0.146 |
|  |  | Pn | -0.879 | 0.460 | 0.125 |
|  |  | Tr | -0.883 | 0.440 | -0.165 |
|  |  | Gs | -0.976 | 0.218 | 0.020 |
|  |  | FvFm | -0.808 | 0.582 | 0.090 |
|  |  | ASA | -0.501 | 0.824 | 0.265 |
|  |  | APX | -0.794 | 0.118 | -0.597 |
|  |  | DHAR | -0.891 | -0.350 | 0.289 |
|  |  | MDHAR | -0.758 | -0.618 | 0.210 |

**Table 1 Principal component analysis of leaf and root indicators.**
